# Conservative medical treatment cured an interstitial tubal pregnancy complicated by a uterine artery pseudoaneurysm: a case report

**DOI:** 10.3389/fmed.2026.1780037

**Published:** 2026-03-23

**Authors:** Kefang Huan, Di Liu, Zhe Liu, Qianqian Liu, Li Wang, Danni Zhang, Song Han

**Affiliations:** 1 Department of Obstetrics and Gynecology, The 964th Hospital, Changchun, Jilin, China; 2 Department of Pathology, The 964th Hospital, Changchun, Jilin, China

**Keywords:** color Doppler ultrasound, conservative medical treatment, human chorionic gonadotropin, interstitial tubal pregnancy, uterine artery pseudoaneurysm

## Abstract

**Background:**

Uterine artery pseudoaneurysm (UAP) is a localized hematoma that forms after the disruption of the uterine artery wall. Its outer layer is encapsulated by the surrounding tissues and lacks a true vascular wall structure. UAP is a rare complication following uterine arterial injury and often occurs secondary to intrauterine procedures, cesarean section or uterine surgery, and pregnancy. Ultrasound plays an important role in the timely diagnosis of UAP. We present a case of an interstitial tubal pregnancy complicated by UAP identified by ultrasound, which was successfully treated with conservative medication, achieving dual cure.

**Case summary:**

A 23-year-old nulliparous woman presented with “49 days of amenorrhea and 11 days of vaginal bleeding with lower abdominal pain.” This pregnancy followed ovulation induction. Serum human chorionic gonadotropin (*β*-hCG) was 607.3 mIU/mL, with no intrauterine gestational sac seen. She was diagnosed with ectopic pregnancy and started on conservative therapy with 100 mg of oral mifepristone daily. Due to a significant increase in *β*-hCG, on day 5 of treatment, she received 75 mg of intramuscular methotrexate in combination. On day 11, a color Doppler ultrasound revealed a left interstitial tubal pregnancy complicated by UAP (1.0 × 1.3 cm). After the diagnosis, methotrexate was administered again together with ongoing mifepristone, and *β*-hCG began to decline. On day 22, a third round of methotrexate was given; on day 26, the patient suddenly developed lower abdominal pain. An ultrasound showed a mixed echogenic mass in the left interstitial tubal region measuring 3.0 × 3.1 cm, no UAP, and a small amount of pelvic free fluid (1.4 cm). Expectant management was adopted. On day 32, her lower abdominal pain resolved, mifepristone was discontinued, and she was discharged. Complete blood count (CBC), liver function, and renal function were monitored throughout and remained normal. During conservative therapy with mifepristone, *β*-hCG fluctuated between 607.3 and 2,375 mIU/mL. Through three rounds of methotrexate combined with continuous mifepristone treatment, dual clinical cure of interstitial tubal pregnancy and UAP was achieved. A 6-month follow-up showed no recurrence of UAP in the left interstitial tubal area and normal serum *β*-hCG.

**Conclusion:**

In interstitial tubal pregnancy complicated by UAP, ultrasound has important diagnostic value for UAP. A conservative medical regimen can have dual therapeutic effects on both the interstitial pregnancy and the UAP.

## 1 Introduction

**Figure 1:**
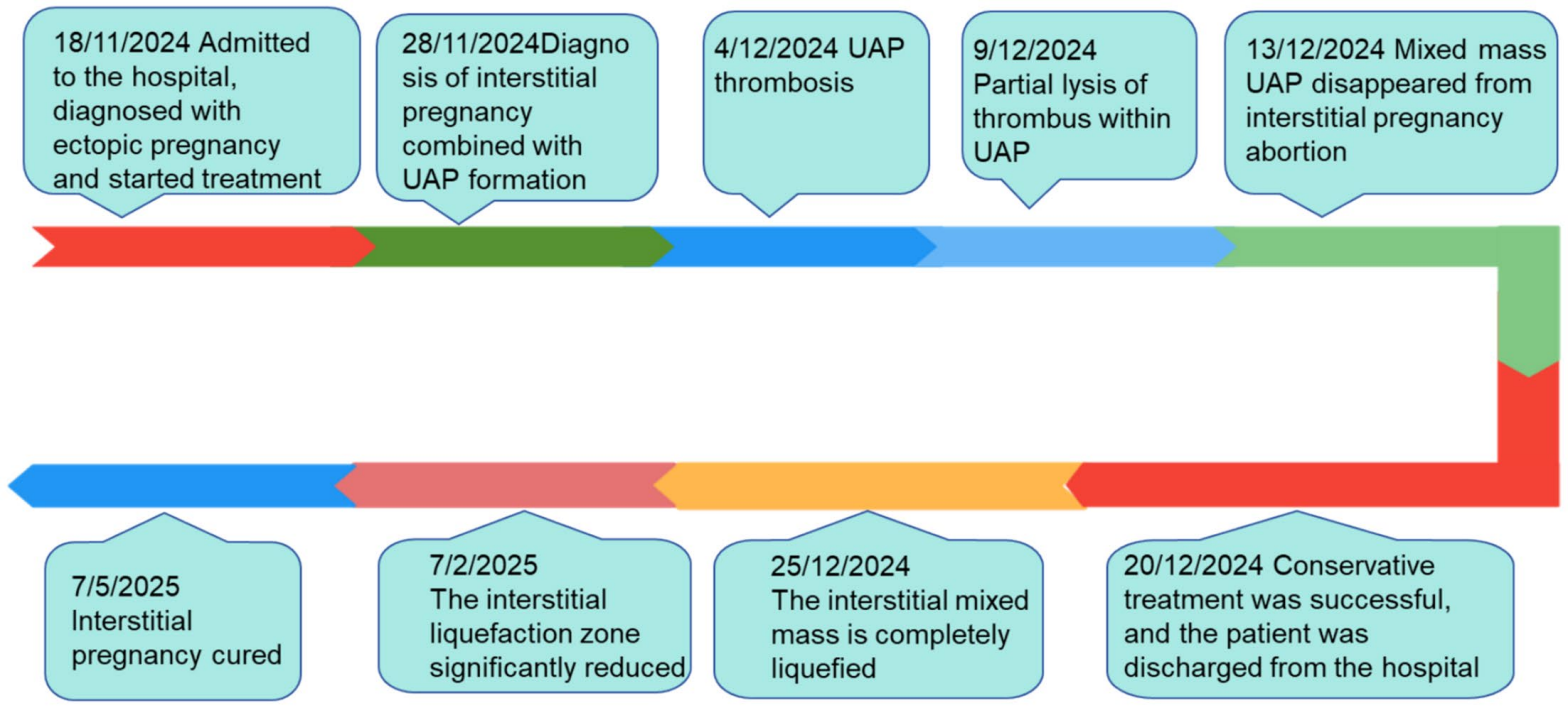
Timeline of patient diagnosis and treatment.

Uterine artery pseudoaneurysm (UAP) may rupture and bleed, potentially becoming life-threatening (1–6). Early diagnosis and intervention is necessary. Interstitial tubal pregnancy complicated by UAP increases bleeding risk, and ultrasound can effectively improve diagnostic accuracy for interstitial pregnancy with UAP to aid precise clinical management. In this article, we describe in detail a successfully managed case of interstitial tubal pregnancy with UAP treated conservatively with medication (Figure 1).

## 2 Case presentation

**Figure 2:**
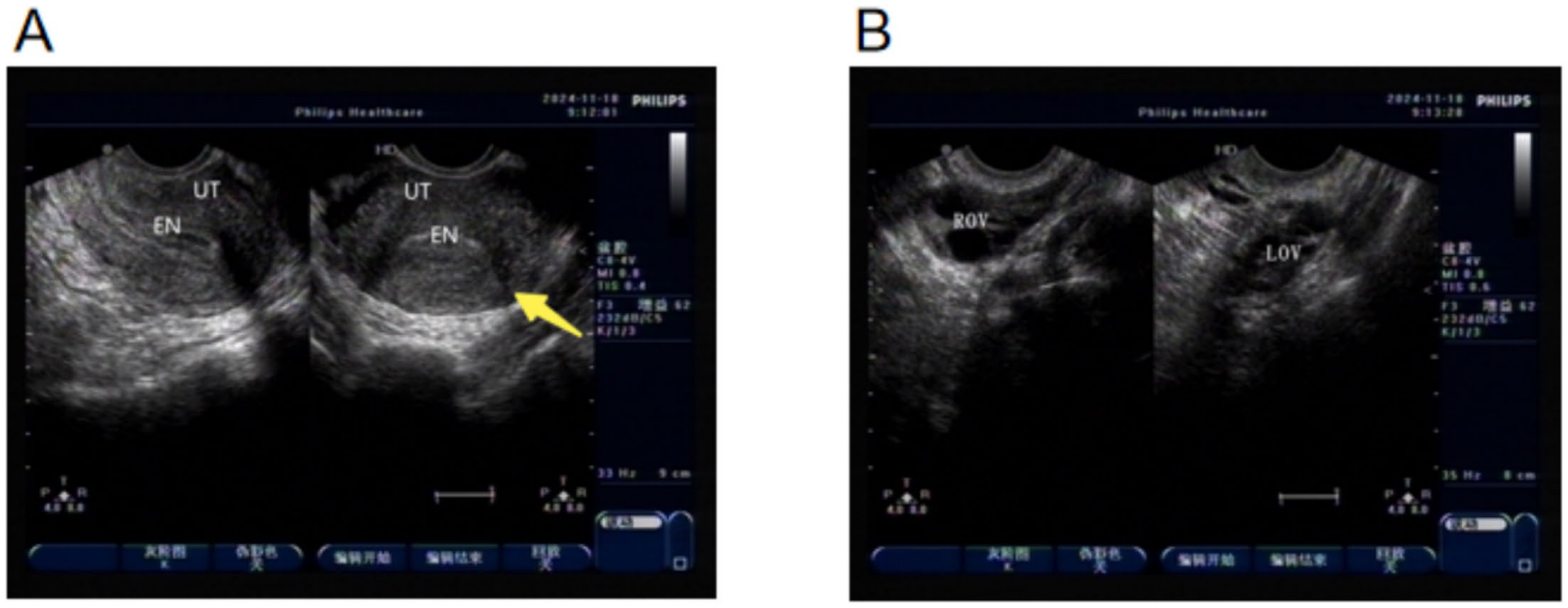
Ultrasound images on the day of admission. **(A)** Yellow arrow: no pregnancy lesion seen in the left interstitial tubal region. **(B)** Bilateral ovaries.

A 23-year-old married nulliparous woman presented with “49 days of amenorrhea and 11 days of vaginal bleeding with lower abdominal pain.” The pregnancy followed ovulation induction. On day 30 of amenorrhea, she had vaginal bleeding with cramping lower abdominal pain and a sense of rectal pressure. On days 34–42 of amenorrhea, serum *β*-hCG increased from 46.19 mIU/mL on the first day of admission to 607.3 mIU/mL. An ultrasound showed no intrauterine gestational sac. The clinical diagnosis was ectopic pregnancy. She was given 100 mg of oral mifepristone daily as conservative therapy. Five days after treatment, serum *β*-hCG increased to 1,837 mIU/mL, and 75 mg of intramuscular methotrexate was administered, with continued mifepristone at the original dose (Figure 2).

**Figure 3:**
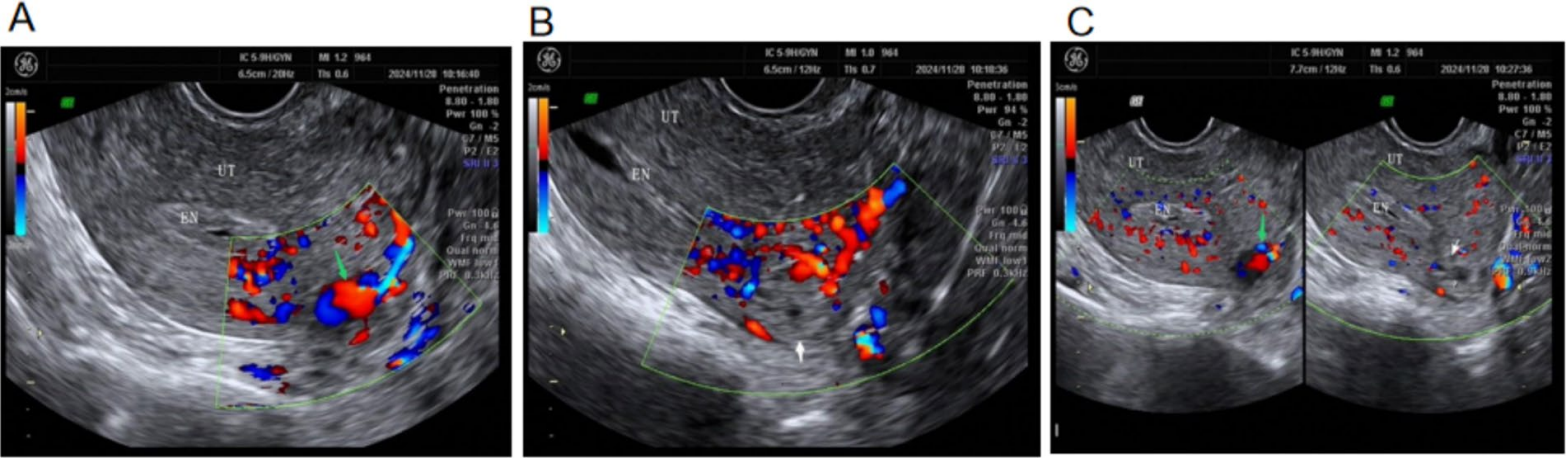
Ultrasound images on day 11 of conservative treatment. **(A)** Green arrow: UAP formation with vortex sign. **(B)** White arrow: left interstitial tubal pregnancy lesion. **(C)** Spatial relationship between the interstitial pregnancy lesion and the UAP.

On day 11, serum β-hCG was 2,137 mIU/mL. An ultrasound showed that the endometrium was 9-mm thick, with no gestational sac seen in the uterus; a 1.0 × 1.0 cm-cystic echo was visible on the left side of the uterine fundus; the endometrium was discontinuous; a fine echo line was visible between the endometrium and the cyst at the uterine horn, presenting as the “interstitial line sign” (7, 8); the myometrium around the cyst was thin, measuring approximately 1 mm; a raised serosa layer was visible on the upper outer side of the fundus; blood flow was seen around the cyst; and a 1.0 × 1.3-cm anechoic area was seen inside. Color doppler flow imaging (CDFI) demonstrated a uterine arterial inflow entering the anechoic area, with intermingled red–blue signals and a vortex pattern. Ultrasound diagnosis showed left interstitial tubal pregnancy with UAP (Figure 3). Uterine cavity fluid accumulation was observed. The patient declined surgery. Repeat testing showed normal CBC, liver, and renal function. A total of 75 mg of intramuscular methotrexate was given with continued oral mifepristone. On day 15, *β*-hCG decreased to 1,680 mIU/mL.

On day 17, an ultrasound showed no gestational sac in the uterus; the interstitial mass decreased to 0.9 × 0.4 cm with peripheral blood flow; and the UAP reduced to 0.8 × 0.8 cm and displayed heterogeneous isoechoic content. A uterine arterial inflow was seen entering it; the internal vortex sign had disappeared, indicating thrombus formation within the UAP. Uterine cavity fluid was seen. On day 18, *β*-hCG decreased to 1,006 mIU/mL, and 100 mg of mifepristone was continued orally once daily.

**Figure 4:**
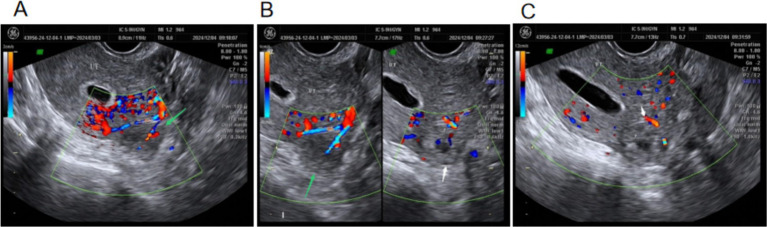
Ultrasound images on day 17 of conservative treatment. **(A)** Thrombus formation within the UAP. **(B)** Coexistence of UAP and left interstitial tubal pregnancy. **(C)** Reduced blood flow within the left interstitial pregnancy lesion.

On day 22, β-hCG dropped to 884.9 mIU/mL. An ultrasound suggested left interstitial tubal pregnancy with partial thrombus dissolution within the UAP. On examination, mild lower abdominal tenderness was present; CBC, liver, and renal function were normal. 75 mg of intramuscular methotrexate was administered again together with mifepristone at the original dose (Figure 4).

**Figure 5:**
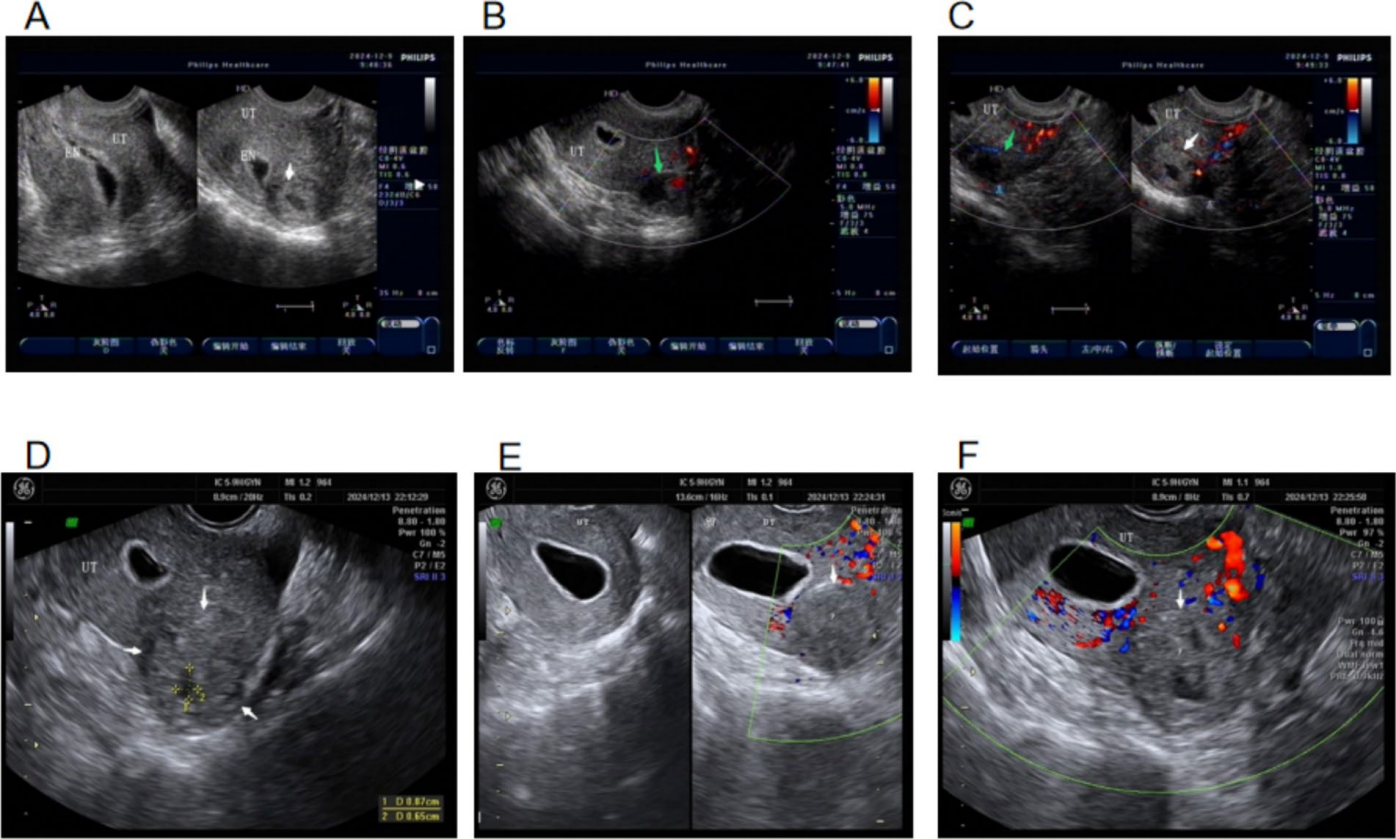
Ultrasound on days 22 and 26 of treatment. **(A)** White arrow: left interstitial tubal pregnancy. **(B)** Green arrow: partial thrombus dissolution within UAP. **(C)** UAP and left interstitial tubal pregnancy. **(D, E)** White arrows: mixed mass formed by miscarriage of the left interstitial tubal pregnancy; UAP disappeared. **(F)** White arrow: local magnified view of the mixed mass formed by miscarriage of the left interstitial tubal pregnancy.

On day 26, the patient developed sudden lower abdominal pain. An ultrasound showed that the endometrial thickness was 4 mm, and the uterine cavity was separated with an 18-mm-thick endometrial fluid area. No gestational sac was seen in the uterus; a 3.0 × 3.1-cm mixed echogenic mass was visible on the left side of the uterine fundus, discontinued from the endometrium; a fine echoic line, resembling the “interstitial line sign,” was visible between the endometrium and the cyst in the uterine horn; and no myometrial tissue was seen around the cyst, but a raised serosa was visible on the outer side of the uterine fundus. CDFI showed no internal blood flow, with punctate peripheral signals. The ultrasound showed a left interstitial tubal pregnancy without UAP. A small amount of pelvic fluid (1.4 cm) was present, suggesting separation of the interstitial tubal gestational tissue from the tubal wall with bleeding (Figures 5D–F). On day 27, *β*-hCG dropped to 608.9 mIU/mL, and lower abdominal pain lessened. A total of 50 mg of mifepristone was continued orally once daily.

**Figure 6:**
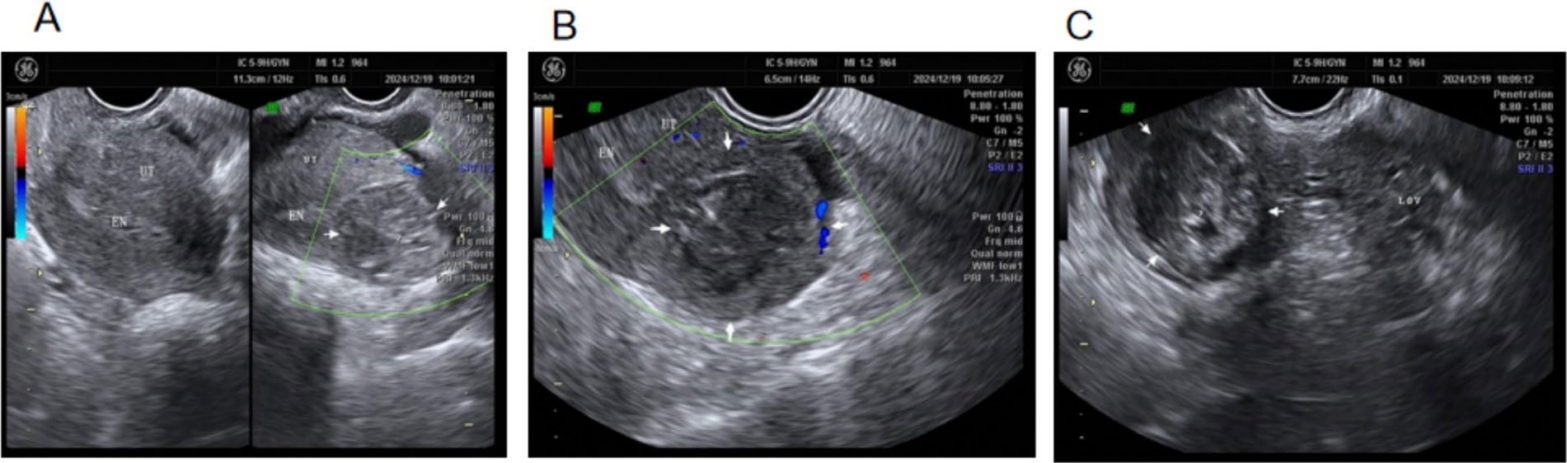
Slight enlargement of the mixed mass in the left interstitial tubal region after conservative medical therapy.

On day 32, the patient’s lower abdominal pain resolved; serum β-hCG dropped to 26.95 mIU/mL. An ultrasound showed a mixed echogenic mass in the left interstitial area measuring approximately 3.8 × 2.8 cm, partially protruding externally, without internal blood flow (Figure 6). Mifepristone was discontinued, and the patient was discharged.

**Figure 7:**
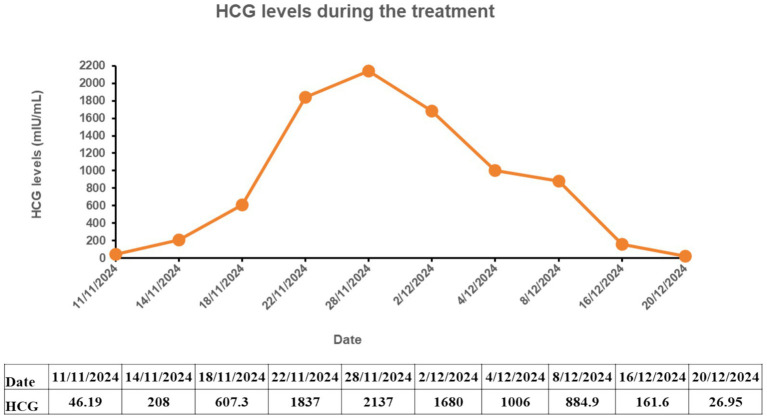
Changes in *β*-hCG during conservative medical treatment.

**Figure 8:**
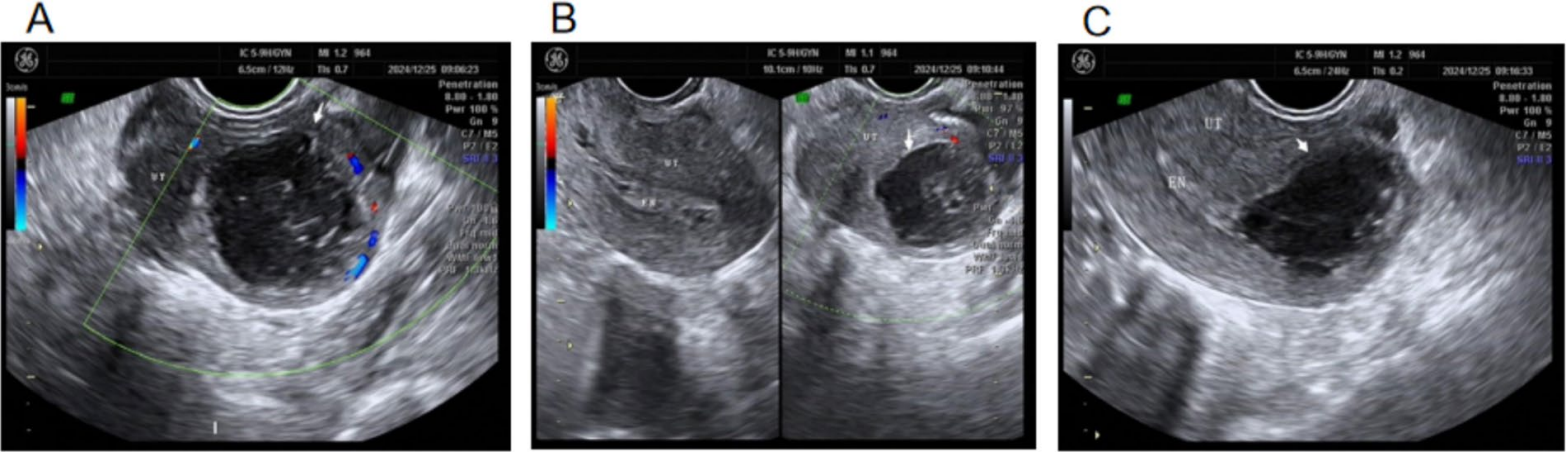
Gynecologic ultrasound images on 5 days after discharge. **(A–C)** White arrows: necrosis and liquefaction of the left interstitial tubal pregnancy connected to the uterine cavity.

During conservative medical treatment, *β*-hCG decreased from 1837 mIU/mL to 26.95 mIU/mL (Figure 7). The lower abdominal pain resolved, conservative treatment was successful, and the patient was discharged. On day 5 post-discharge, a follow-up ultrasound showed an anechoic area of approximately 3.0 × 3.1 cm in the left cornual and interstitial tubal region, with irregular shape, poor acoustic through-transmission, and no internal blood flow (Figure 8). Two weeks after discharge, *β*-hCG was normal.

**Figure 9:**
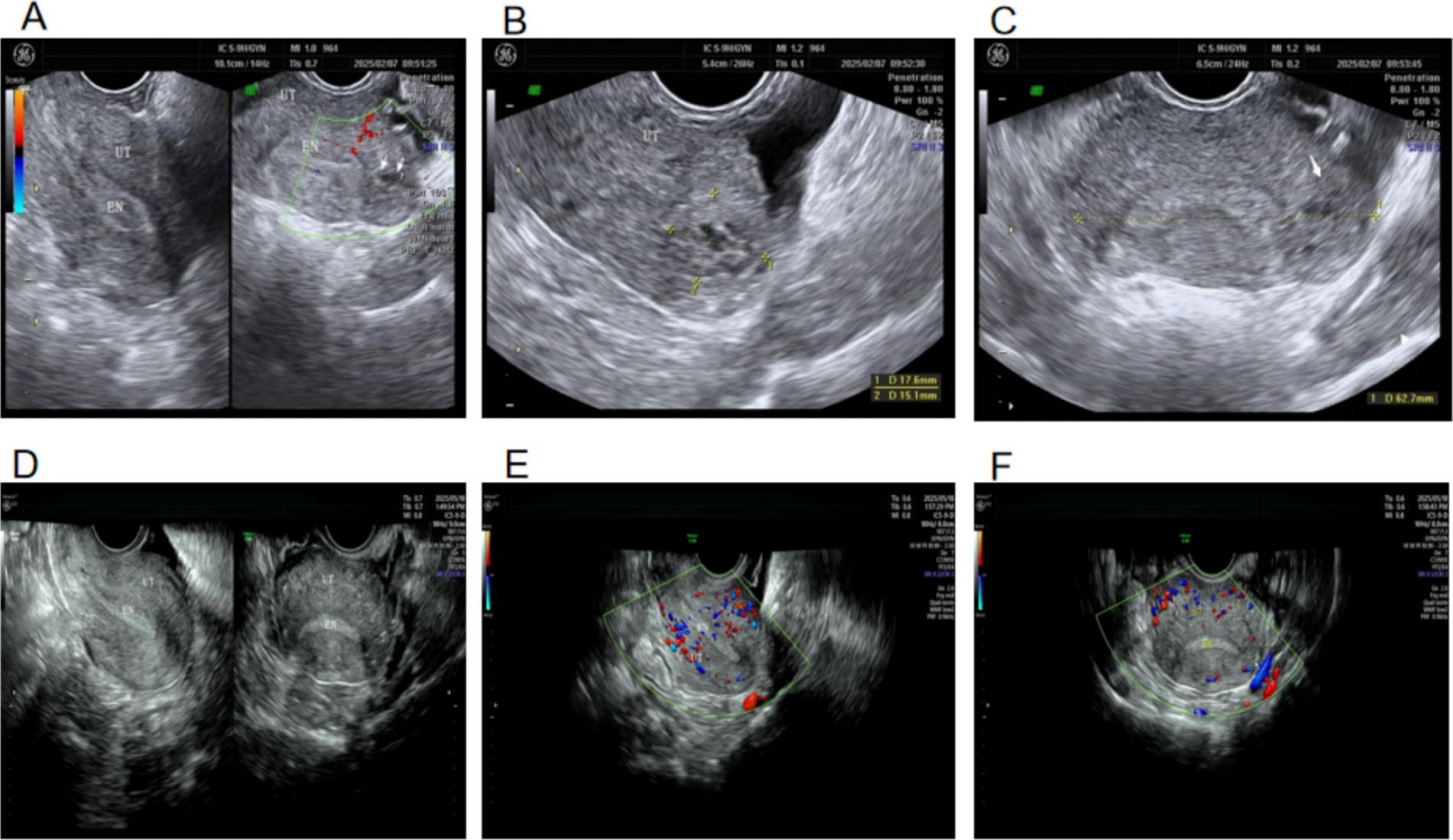
Gynecologic ultrasound images on days 49 and 138 after discharge. **(A)** White arrow: significant reduction of the liquefied area in the left interstitial tubal region. **(B)** Local schematic of liquefaction in the left interstitial tubal pregnancy area. **(C)** Transverse section of the liquefied area in the left interstitial tubal region. **(D)** The fluid area in the left interstitial tubal region has been absorbed. **(E)** Longitudinal section of the uterus. **(F)** Transverse section of the uterus.

On day 18 after discharge, a transvaginal ultrasound showed an anechoic area of approximately 1.8 × 1.5 cm in the left cornual and left interstitial tubal region with poor acoustic through-transmission, suggesting fluid collection in the left cornual and left interstitial tubal area (Figures 9A–C). At 6 months after discharge, a transvaginal ultrasound showed no abnormalities in the left interstitial tubal region (Figures 9D–F).

## 3 Discussion

The key point in this case lies in the timely diagnosis of UAP during the treatment of interstitial tubal pregnancy. Color Doppler ultrasound is of great value in the diagnosis of UAP. UAP is a rare uterine vascular complication, typically resulting from iatrogenic injury, trauma, or inflammation leading to partial rupture of the arterial wall, with blood extravasation encapsulated by surrounding tissues (9). Unlike true aneurysms, UAP lacks a complete tri-layered arterial wall and consists only of a single layer of loose connective tissue. Repeated arterial blood flow impact easily causes the aneurysm wall to rupture and bleed (10). UAP happens most commonly after cesarean section, with an incidence of approximately 0.2–1% (11). It has occasionally been reported in association with trophoblastic disease and infection, which are related to injury or rupture of the uterine arterial wall (12, 13). In this case, UAP occurred during conservative medical management, consistent with a complication of medical abortion.

Reports indicate that ultrasound diagnostic sensitivity for UAP is approximately 89%, with a specificity of up to 95% (14, 15). Hemodynamic ultrasound features of UAP mainly include: (1) a “to-and-fro” flow pattern at the neck of the sac (arterial blood shoots into the sac through the neck at high speed during systole and then reverses to flow back into the uterine artery during diastole), (2) intermingled red–blue vortex signals within the sac (vortex flow pattern), and (3) the typical “yin-yang sign” (16, 17). On the 11th day of treatment in this case, a color Doppler ultrasound showed typical imaging features of left interstitial tubal pregnancy and UAP. Ultrasound leveraged the hallmark feature of a pseudoaneurysm sac communicating with the injured artery through a narrow neck to achieve a timely diagnosis. The mechanism here likely relates to local embryonic tissue necrosis after conservative treatment of interstitial pregnancy, causing arterial wall injury and rupture (8).

The main challenge in this case was selecting the treatment strategy after confirming interstitial tubal pregnancy with UAP. First, we considered whether the late detection of interstitial pregnancy was related to conservative management. Some literature reports that interstitial pregnancy can be diagnosed as early as 6.9–8.2 weeks (18). The normal implantation time for a fertilized egg should be between 22 and 26 days after the last menstrual period. Based on the medical history, early examination results from other hospitals, and ovulation induction, it is estimated that the implantation had already occurred. The ultrasound image did not show a positive result, but the patient had already experienced abdominal pain and vaginal bleeding, raising suspicion of an ectopic pregnancy. To prevent rupture and disease progression, drug intervention was administered. As this case was a retrospective analysis, early intervention at this time was insufficient, and further magnetic resonance imaging was needed to confirm the location of pregnancy. We inferred an ectopic pregnancy based solely on clinical symptoms and ovulation induction time. Literature has reported successful experiences with mifepristone in treating ectopic pregnancy, with daily doses of 50–300 mg (19–22). The duration of treatment depended on changes in the patient’s condition. A meta-analysis reported that the average duration of conservative treatment for ectopic pregnancy was 42–50 days, with the longest treatment duration reaching 84 days (23). Furthermore, there are literature reports of successful treatment of interstitial pregnancy with mifepristone (24); thus, we chose mifepristone to treat ectopic pregnancy. Literature reports that methotrexate combined with mifepristone is more effective than single-drug therapy (22). Therefore, given that the decrease in *β*-hCG was not ideal on day 5 of treatment, we added methotrexate treatment. However, when interstitial pregnancy with UAP was confirmed on day 11, the treatment plan needed revision. On day 5 of conservative therapy, the patient’s β-hCG had increased to triple the admission value, prompting combination treatment with methotrexate. Arterial embolization is considered the first-line treatment for UAP (25, 26). Patients with stable vital signs, scant vaginal bleeding, and sacs with a long axis of <5 cm may be suitable for conservative treatment (27). In this patient, the UAP sac was <5 cm but secondary to interstitial tubal pregnancy. Treatment options for interstitial pregnancy include surgery, surgery after embolization, and conservative therapy (28, 29). In this case, given the inadequate decline in *β*-hCG with conservative therapy and the coexistence of UAP, the risk of rupture and hemorrhage was high (30). Based on the diagnosis, post-embolization surgery should be selected at this time (26). After discussing with the patient and her family, uterine artery embolization followed by resection of the right cornua and pseudoaneurysm was recommended. The patient, who had a strong desire for fertility, chose conservative medical treatment and declined surgery after fully informed consent. Considering that *β*-hCG was 2,137 mIU/mL with a downward trend compared to the prior value, the patient showed stable vital signs, and lower abdominal tenderness had not worsened. Methotrexate was added again, and mifepristone was maintained. Following this, the *β*-hCG decline was satisfactory. Another challenge was encountered on day 26 when the patient developed persistent severe lower abdominal pain, requiring assessment for possible rupture of the interstitial pregnancy or UAP necessitating emergency surgery. There are reports of massive hemorrhage due to interstitial rupture during conservative management (28). In our analysis, β-hCG showed a marked downward trend; ultrasound indicated the interstitial mass enlarged to 3.0 × 3.1 cm with 1.4 cm of pelvic fluid, suggesting that the pain might be due to local embryonic tissue necrosis and detachment causing mild pelvic effusion, rather than rupture of the interstitial pregnancy or UAP. Prior to this, thrombosis had already formed within the UAP. Comprehensive evaluation led to expectant management with readiness for urgent surgery if deterioration occurred. Fortunately, the abdominal pain was short-lived, and no interstitial rupture or UAP rupture occurred. The treatment achieved the expected results, and the patient was discharged after recovery. Mifepristone was continued throughout, with three doses of methotrexate added; given the low dosing, folinic acid rescue for methotrexate toxicity was not used (31).

In summary, in interstitial tubal pregnancy complicated by UAP, diagnosis relies on characteristic ultrasound findings. The focus is often on the ectopic pregnancy, potentially overlooking UAP; the coexistence of both is rare. With close monitoring of symptoms, signs, *β*-hCG, CBC, liver and renal function, mass size, and UAP status, methotrexate combined with ongoing mifepristone can achieve dual cure of interstitial pregnancy and UAP.

## Data Availability

The original contributions presented in the study are included in the article/supplementary material, further inquiries can be directed to the corresponding authors.
